# Investigating Cesarean Section Trends: A Study Using the Modified Robson Classification in a Tertiary Care Setting

**DOI:** 10.7759/cureus.72827

**Published:** 2024-11-01

**Authors:** Hiba Arshad, Zahid H Wadani, Zunaira Kamal, Shazia Masheer

**Affiliations:** 1 Obstetrics and Gynaecology, Aga Khan University Hospital, Karachi, PAK

**Keywords:** caesarean section rate, ecv, induction of labor, robson classification, vaginal birth after cesarean (vbac)

## Abstract

Introduction: The cesarean section (CS) rate is steadily rising in developed and developing nations. While CS is often perceived as a life-saving intervention, it comes with associated dangers for both the present and future pregnancies. The Robson criteria classify CS into 10 groups with further sub-divisions based on the gestational age, various pregnancy categories, prior obstetrical records, and the progress of labor and delivery.

Methods: This retrospective record review was conducted at Aga Khan University Hospital in Karachi, Pakistan. A non-probability consecutive sampling method was employed to enroll all 1198 pregnant women who underwent CS delivery between July 1st and December 31st, 2022.

Results: A total of 2616 deliveries were performed during the study period; among these, 1198 (45%) were delivered by CS. Out of 10 groups of Robson Criteria, Group 5 emerged as the primary contributor to the overall CS rate, making up approximately 34.3% of cases, followed by Group 10, which contributed to 28.5% of cases. Group 2 contributed 16.4% of cases in total, with subgroup 2A constituting 12% of the overall rates, and Group 1 was found to be the fourth contributing category (5.1%), succeeded by Group 6 (4.9%), Group 4 (3.9%), and Group 8 (3.4%).

Conclusion: The Modified Robson Criteria was found to be an effective and easy-to-comprehend tool, which assists in monitoring the indications of CS and aids in devising strategies to reduce its rates in clinical practice.

## Introduction

Cesarean section (CS) rates can successfully prevent maternal and perinatal mortality and morbidity when medically necessary. World Health Organization (WHO) proposed an ideal CS rate of 10-15% in 1985 [1-3]. Worldwide, for a variety of reasons, the CS rate is steadily rising in both developed and developing nations [4]. CS is thought to be a life-saving intervention, but there are dangers associated with it for current and future pregnancies. In obstetrics, it has brought about an unanticipated transformation that would eventually result in a rise in maternal morbidity and mortality. Despite its advantages, it is important to remember that there are risks involved, including infection, hemorrhage, anesthesia complications, and the well-known placental spectrum disorder [5]. Avoiding CS may not help in situations where spontaneous vaginal delivery is either impossible or dangerous and may lead to distressful situations for both the mother and the fetus [6]. Maternal morbidity, including more blood transfusions, uterine scar rupture, placental spectrum disorder, placenta previa, and hysterectomies, are all linked to the increase in CS rates [7].

Based on two multi-country surveys, the WHO has officially recognized and recommended Robson's 10-group classification system, proposed by Dr. Michael Robson in 2001, as a global standard for evaluating, monitoring, and comparing CS rates within healthcare facilities [8]. The "Ten Group Classification System" divides CS into 10 groups based on the gestational age of the pregnancy, various pregnancy categories, prior obstetrical records, and the progress of labor and delivery [6,9]. The Society of Obstetrics and Gynecology of Canada modified the categorization subsequently. This change subclassifies women who underwent CS before labor, after labor is induced, and into spontaneous labor [7].

The key challenge in the current situation is to keep the CS rate as low as possible while ensuring the safety of both the mother and the newborn. This can be done by reviewing/auditing CS rates performed in medical facilities at regular intervals. As frameworks for auditing, the three most frequently used classifications are based on “primary clinical indications," "the degree of urgency or absolute need for CS delivery," and "Robson classification" [6]. In a systematic analysis comparing various classifications for CS, Torloni et al. concluded that Robson's 10 Groups classification was most appropriate for monitoring CS rates [10].

The study was conducted at our healthcare center, recognized for its expertise in managing complex obstetric cases across the country. Our center is equipped with advanced resources, including a blood bank and highly skilled surgeons specialized in treating placental spectrum disorders. Additionally, the state-of-the-art neonatal intensive care Unit (NICU) facility is also available. Previous audits at our center distinguish between primary emergency and repeated CS. The in-depth analysis of causes leading to CS was lacking. The objective of this study was to identify the key factors for performing CS using modified Robson criteria. This would help in developing targeted strategies to reduce the CS rate.

## Materials and methods

This retrospective record review was conducted at Aga Khan Hospital in Karachi, Pakistan. All pregnant women with gestation greater than 26 weeks who underwent CS during July 1st and December 31st, 2022 were considered eligible to be included in the study. A non-probability consecutive sampling method was employed to enroll the participants after obtaining approval from the Aga Khan University Hospital (AKUH) Ethics Review Committees (ERC: 2022-8272-23687). Patient information, including age, gravida, parity, conception, gestational age at delivery, fetal birth weight, and fetal presentation, among other variables, was gathered from hospital records using a predefined questionnaire within the hospital's information management system. CS-delivered women were categorized according to the modified Robson criteria and divided into spontaneous, induced, and pre-spontaneous labor groups based on labor onset.

Statistical analysis

Data analysis was performed using Statistical Package for Special Sciences (SPSS) version 20 (SPSS; IBM Corporation, Armonk, NY). Descriptive statistics were computed for all the variables. Frequencies and percentages were computed for qualitative variables, while means and standard deviations were computed for quantitative variables. Group-specific distribution of CS rate according to Robson criteria has been expressed as frequencies and percentages.

## Results

During the study period, the CS rate constituted around 1198 (45%) of all 2616 deliveries performed. Around 751 (62.7%) and 447 (37.3%) women were multigravida and primigravida, respectively. Around 642 (53.6%) women had a history of previous CS. The majority of women conceived spontaneously, i.e., 1158 (97.7%), with only 40 (96.7%) having a history of miscarriage. The mean gestational age of the fetus at the time of delivery was around 36.42±2.4 weeks with the majority presenting in cephalic position, i.e., 1084 (90.5%). The maternal and fetal characteristics are shown in Table 1.

**Table 1:** Fetal and maternal characteristics of women who delivered via CS (n=1198) The data has been presented as frequency (percentages) and mean±standard deviation. IVF, in vitro fertilization; ICSI, intracytoplasmic sperm injection; GDM, gestational diabetes mellitus; PIH, pregnancy-induced hypertension; IUGR, intrauterine growth restriction; CS, cesarean section

S.no.	Characteristics	Frequency (%)	Mean ±SD
1	Maternal age (years)	-	31.6±5.15
2	Gravida	-	2.12±1.14
	1	447 (37.3%)	-
	2	365 (30.5%)	-
	3	227 (18.9%)	-
	4	123 (10.3%)	-
	>4	36 (3%)	-
3	Parity	-	1.04±1.06
	0	456 (38.1%)	-
	1	395 (33.0%)	-
	2	221 (18.4%)	-
	≥3	126 (10.5%)	-
4	History of miscarriage	-	-
	0	1158 (96.7%)	-
	1	14 (1.2%)	-
	>1	26 (2.17%)	-
5	Conception methods	-	-
	Spontaneous	1171 (97.7%)	-
	IVF	18 (1.5%)	-
	ICSI	2 (0.2%)	-
	Ovulation induction	7 (0.6%)	-
6	Gestational age (weeks)	-	36.42±2.40
7	History of CS	642 (53.6%)	-
8	Spontaneous onset of labor	229 (19.1%)	-
9	Fetal presentation	-	-
	Cephalic	1084 (90.5%)	-
	Breech	94 (7.8%)	-
	Abnormal lie/twins/triplets	20 (1.66%)	-
10	Apgar score (at 5 minutes)	-	-
	>7	1194 (99.7%)	-
	≤7	4 (0.3%)	-
11	Birth weight (kg)	-	2.67±0.59
12	Co-morbid	-	-
	GDM	450 (37.6%)	-
	Hypothyroidism	73 (6.1%)	-
	Pre-eclampsia	227 (18.9%)	-
	PIH	104 (8.7%)	-
	IUGR	74 (6.2%)	-
	Obstetric cholestasis	71 (5.9%)	-
	Others	290 (24.2%)	-

The CS rate within each 10 groups of Robson criteria was analyzed to understand its specific impact on the overall CS rate. As illustrated in Table 2, Group 5 emerged as the primary contributor to the overall CS rate, making up approximately 34.3%, notably with subgroup 5C showing the highest percentage at 30.9%. The second significant category influencing the CS rate was Group 10, contributing to an overall percentage of 28.5%, with subgroup 10C accounting for about 18.5% of cases. Following closely was Group 2, contributing 16.4% in total, with subgroup 2A constituting 12% of the overall CS rates. Subsequently, the fourth contributing category was Group 1 (5.1%), succeeded by Group 6 (4.9%), Group 4 (3.9%), and Group 8 (3.4%). This was followed by Group 7 (all multiparous breaches, including the previous scar) with 2.2% of all. 

**Table 2:** Group-specific distribution of CS deliveries among women The data has been presented as frequency (percentages). CS, cesarean section

Group	Description	Sub-group contribution	Overall contribution
1	Nulliparous, singleton cephalic, ≥37 weeks, in spontaneous labor	61 (5.1%)	5.1%
2	Nulliparous, singleton cephalic, ≥37 weeks	-	16.4%
2A	Induced	144 (12%)	-
2B	CS before labor	53 (4.4%)	-
3	Multiparous, single cephalic, ≥37 weeks, spontaneous labor	6 (0.5%)	0.5%
4	Multiparous, single cephalic, >37 weeks	-	3.9%
4A	Induced	30 (2.5%)	-
4B	CS before labor	17 (1.4)	-
5	Previous CS, single cephalic, ≥37 weeks	-	34.3%
5A	Spontaneous labor	39 (3.3%)	-
5B	Induced labor	1 (0.1%)	-
5C	CS before labor	370 (30.9%)	-
6	All nulliparous breeches	-	4.9%
6A	Spontaneous labor	5 (0.4%)	-
6B	Induced labor	8 (0.7%)	-
6C	CS before labor	46 (3.8%)	-
7	All multiparous breeches (including previous CS)	-	2.2%
7A	Spontaneous labor	9 (0.8%)	-
7B	Induced labor	0	-
7C	CS before labor	17 (1.4%)	-
8	All multiple pregnancies	-	3.4%
8A	Spontaneous labor	9 (0.8%)	-
8B	Induced labor	1 (0.1%)	-
8C	CS before labor	30 (2.5%)	-
9	All abnormal lies (including previous CS but excluding breech)	-	0.9%
9A	Spontaneous labor	1 (0.1%)	-
9B	Induced labor	3 (0.3%)	-
9C	CS before labor	6 (0.5%)	-
10	All singleton cephalic, ≤36 weeks (including previous CS)	-	28.5%
10A	Spontaneous labor	106 (8.8%)	-
10B	Induced labor	14 (1.2%)	-
10C	CS before labor	222 (18.5%)	-

Table 3 presents the reasons for CS within each group. The leading reason for CS was a previous scar, accounting for 34 (89%) and 361 (99%) cases in Group 5A and Group 5C, respectively, and approximately 58 (56%) cases in Group 10. Non-progress of labor constituted about 28 (48%) and 87 (62.1%) of cases in Group 1 and Group 2A, respectively. Fetal distress was observed in around 23 (39%) and 27 (19%) of cases in Group 1 and Group 2A, respectively. Additionally, 12 (71%) cases in Group 4B and 22 (42%) cases in Group 2B were due to patient requests or denial of the trial of labor. Breech abnormal lies contributed to approximately 32 (71%) cases in Group 6C. Other reasons such as fetal growth restriction, suspicious cardiotocography (CTG) trace, severe medical disorders, pre-eclampsia, twin pregnancy, antepartum hemorrhage, preterm complications, and placenta previa also contributed to the CS rate.

**Table 3:** Group-specific reasons for CS deliveries among women The data has been presented as frequency (percentage). CS, cesarean section

Groups	Breech abnormal lie	Fetal growth restriction	Non-progress of labor	Fetal distress	Patients’ request	Previous scar	Severe medical disorder	Pre-eclampsia	Others	Total
1	-	-	28 (48%)	23 (39%)	6 (10%)	-	-	-	2 (4%)	59
2	-	-	-	-	-	-	-	-	-	-
2A	4 (3%)	-	87 (62%)	27 (19%)	19 (14%)	-	-	1 (0.1%)	2 (1.4%)	140
2B	-	-	2 (4%)	16 (31%)	22 (42%)	2 (4%)	2 (4%)	1 (2%)	7 (13%)	52
3	-	-	-	3 (75%)	-	-	-	-	1 (25%)	4
4	-	-	-	-	-	-	-	-	-	-
4A	-	-	15 (50%)	13 (43%)	-	-	-	-	2 (7%)	30
4B	-	-	-	-	12 (71%)	2 (12%)	-	-	3 (18%)	17
5	-	-	-	-	-	-	-	-	-	-
5A	-	-	3 (8%)	1 (3%)	-	34 (89%)	-	-	-	38
5B	-	-	-	-	-	1 (100%)	-	-	-	1
5C	-	-	-	1 (0.3%)	-	361 (99%)	-	-	1 (0.3%)	363
6	-	-	-	-	-	-	-	-	-	-
6A	4 (100%)	-	-	-	-	-	-	-	-	4
6B	8 (18%)	-	-	-	-	-	-	-	-	8
6C	32 (71%)	4 (9%)	-	-	-	-	9 (20%)	-	-	45
7	-	-	-	-	-	-	-	-	-	-
7A	8 (89%)	-	-	-	-	-	-	1 (11%)	-	9
7B	-	-	-	-	-	-	-	-	-	0
7C	12 (71%)	-	-	-	-	4 (24%)	-	1 (6%)	-	17
8	-	-	-	-	-	-	-	-	-	-
8A	-	1 (13%)	-	-	-	5 (63%)	-	-	2 (25%)	8
8B	-	-	1 (100%)	-	-	-	-	-	-	1
8C	7 (25%)	-	-	6 (21%)	-	6 (21%)	-	-	9 (32%)	28
9	-	-	-	-	-	-	-	-	-	-
9A	-	-	-	-	-	-	-	-	-	-
9B	3 (100%)	-	-	-	-	-	-	-	-	3
9C	4 (80%)	-	-	-	-	-	-	-	1 (20%)	5
10	-	-	-	-	-	-	-	-	-	-
10A	-	1(1%)	13(13%)	7 (7%)	-	58 (56%)	-	4 (4%)	21 (20%)	104
10B	-	-	2(15%)	6 (46%)	-	-	5 (38%)	-	-	13
10C	-	28(13%)	-	14 (6%)	4 (2%)	82 (38%)	47 (22%)	12 (6%)	30 (14%)	217
Total	82	34	151	117	63	555	63	20	81	1166

## Discussion

The CS rate determined in this study was 45%, aligning closely with rates observed in other private institutions in Pakistan, which typically range from 49% to 56% [11,12]. This study showed higher CS rates in contrast to public sector hospitals where the average rate was around 37% [5]. The higher CS rates may be attributed to the fact that it is a tertiary care hospital where most complicated pregnancies are referred.

Compared to other groups, Group 5 exhibited the highest CS rates (34.3%), within this subgroup 5C having the highest rates at 30.9%. These results are in concordance with the previous studies conducted in Oman (58.2%) and India (94.49%) [11,13]. The rate of CS in Group 5 from this study was found to be lower compared to other public (56%) and private (80.3%) hospitals in the country [5,7]. Variations in CS rates among hospitals can be attributed to differences in methodologies, knowledge levels, shared risk factors, facilities, and interpretation of results. Reducing these CS rates could involve offering a trial of vaginal birth after cesarean (VBAC) to women with a history of previous CS. Most successful VBAC cases involve advanced cervical dilatation and active labor or prior successful VBAC trials. However, the primary reason for not offering VBAC is the burden of constant supervision of patients, especially in facilities lacking one-to-one care, and the concern about medical-legal issues in case of adverse outcomes. Our institution has a policy for trial after VBAC, with one-to-one nursing care and availability of continuous CTG monitoring, blood bank, 24-hour in-house anesthesia, and obstetric consultant cover. There is a need for the initiation of dedicated VBAC clinics and patient information leaflets to increase awareness and facilitate women's decision-making. Group 10 accounted for the second-highest CS rates (28.5%), within this subgroup 10C (18.5%) contributing the highest within this category. These observations diverge from those of previous studies, where Group 2A was typically identified as the second most prevalent indication for CS, following a history of prior CS [11,5]. The higher prevalence of preterm deliveries at our institute is attributed to our status as a tertiary referral center, boasting state-of-the-art NICU facilities and easy access to highly qualified maternal-fetal medicine experts. The majority of CS is performed in cases of preterm labor with prior uterine scars or severe maternal medical conditions, necessitating multidisciplinary team intervention. These conditions include chronic kidney disease, deteriorating laboratory parameters in type 1 diabetic patients, and exacerbated high-risk autoimmune diseases such as myasthenia gravis and systemic lupus erythematosus.

Subgroup 2A was identified as the third highest contributor, accounting for 12% of CS rates. These results align closely with previous studies conducted in India (11.8%) and Pakistan (12.5%) [5,9]. The primary reasons for CS rates among induced labor patients were predominantly non-progress labor, comprising approximately 62.1%, followed by fetal distress at around 19.2%, and patient refusal for trial or request for CS, accounting for 13.5% within this group. These figures closely mirror the findings of a previous study conducted within the department [14]. This year, a mandatory CTG one-day course for all healthcare staff working in Obstetrics and Gynecology at AKUH has been initiated. This course, facilitated by master trainers trained by UK consultants, aims to enhance expertise in interpreting CTG. This yearly certification of CTG would help in updating staff knowledge and checking the CS done for fetal distress. Additionally, another strategy to lower CS rates in this demographic involves implementing a labor care guide, contrasting with the prevailing practice of adhering solely to the standard partograph [15]. The labor care guide is particularly beneficial for offering one-on-one support, facilitating the presence of a birth companion, and emphasizing the importance of analgesia options. While all these components are in place in our setup, allocating more time for specific dilation of the cervix during active labor would encourage the healthcare team to make more informed decisions.

Lowering the CS rate attributed to nulliparous breech presentations can be achieved by providing an external cephalic version (ECV) to these patients. However, this requires the availability of skilled operators proficient in this maneuver, and institutions must train their doctors, accordingly, considering its approximately 50% success rate. Our institution commenced offering ECV this year, and we have witnessed a favorable success rate. After this study, we are able to categorize and filter down the exact causes of CS and hence able to recommend a few strategies to bring this rate down.

It is important to acknowledge a few limitations of this study. One key limitation is its limited generalizability to larger populations, as it was conducted in a single tertiary care hospital. To gain a more comprehensive understanding of the issue, further studies and audits are needed to monitor practices and CS rates across both urban and rural communities, providing a clearer picture of the true burden.

## Conclusions

The modified Robson's classification offers a comprehensive framework for analyzing laboring women, providing precise insights into labor and delivery characteristics. Its focus on independently assessing primary and repeat CS rates offers clearer, more actionable information. With its straightforward rates, it facilitates the monitoring of CS indications and enables the development of targeted strategies to reduce CS rates. To decrease the repeat CS rates in our center, we aim to target VBAC trials in carefully selected populations. Additionally, there is a pressing need to devise strategies for offering trials to appropriate groups of women with a history of one previous CS. We anticipate a reduction in CS rates following the introduction of yearly training for the entire staff in CTG interpretation and the implementation of ECV for breech presentations.

## References

[1] Organization WH (1985). Appropriate technology for birth. Lancet.

[2] Organization WH (2018). WHO recommendations non-clinical interventions to reduce unnecessary caesarean sections. https://iris.who.int/bitstream/handle/10665/275377/9789241550338-eng.pdf?ua1.

[3] Mascarello KC, Horta BL, Silveira MF (2017). Maternal complications and cesarean section without indication: systematic review and meta-analysis. Rev Saude Publica.

[4] Ansari A, Baqai S, Imran R (2019). An audit of caesarean section rate using modified Robson criteria at a tertiary care hospital. J Coll Physicians Surg Pak.

[5] Majid E, Kulsoom S, Fatima S, Zuberi BF (2022). To evaluate rising caesarean section rate and factors contributing to it by using Modified Robson's Criteria at a tertiary care hospital. Pak J Med Sci.

[6] Parveen R, Khakwani M, Naz A, Bhatti R (2021). Analysis of cesarean sections using Robson’s ten group Classification System. Pak J Med Sci.

[7] Gilani S, Mazhar SB, Zafar M, Mazhar T (2020). The modified Robson criteria for caesarean section audit at Mother and Child Health Center Pakistan Institute of Medical Sciences Islamabad. J Pak Med Assoc.

[8] Robson M, Murphy M, Byrne F (2015). Quality assurance: The 10-Group Classification System (Robson classification), induction of labor, and cesarean delivery. Int J Gynaecol Obstet.

[9] Jayaprakash M (2017). TMC (Thrissur Medical College) modified Robson criteria for caesarean sections. Int J Reprod Contracept Obstet Gynecol.

[10] Torloni MR, Betran AP, Souza JP, Widmer M, Allen T, Gulmezoglu M, Merialdi M (2011). Classifications for cesarean section: a systematic review. PLoS One.

[11] Kazmi T, Saiseema S 5th, Khan S (2012). Analysis of cesarean section rate - according to Robson’s 10-Group classification. Oman Med J.

[12] Uzma S, Kiani B, Khan F (2015). Frequency of placenta praevia with previous caesarean section. Ann Pak Inst Med Sci.

[13] Samba A, Mumuni K (2016). A review of caesarean sections using the ten-group classification system (Robson classification) in the Korle-Bu Teaching Hospital (KBTH), Accra, Ghana. Gynecol Obstet.

[14] Dur-E-Shahwar Dur-E-Shahwar, Ahmed I, Amerjee A, Hoodbhoy Z (2018). Comparison of neonatal outcomes between Category-1 and non-category-1 primary emergency cesarean section: a retrospective record review in a tertiary care hospital. Pak J Med Sci.

[15] Patabendige M, Wickramasooriya DJ, Dasanayake DL (2021). WHO Labor Care Guide as the next generation partogram: revolutionising the quality of care during labor. Eur J Midwifery.

